# An ethnopharmacological study of aromatic Uyghur medicinal plants in Xinjiang, China

**DOI:** 10.1080/13880209.2016.1270971

**Published:** 2017-02-16

**Authors:** Lu Zhao, Shuge Tian, E. Wen, Halmuart Upur

**Affiliations:** a College of Chemistry and Chemical Engineering, Xinjiang Normal University, Urumqi, Xinjiang, China;; b Central Laboratory of Xinjiang Medical University, Urumqi, Xinjiang, China;; c College of TCM, Xinjiang Medical University, Urumqi, Xinjiang, China

**Keywords:** Ethnic, herbs, traditional, volatile oil, use value

## Abstract

**Context:** An ethnobotanical survey was completed in a remote village and surrounding country of Xinjiang, where most Uyghur medicinal plants could be collected. This work clarifies and increases ethnobotanical data.

**Objectives:** We surveyed and organized aromatic medicinal plants that are commonly used in clinical settings to provide a significant reference for studying new medical activities.

**Materials and methods:** In the survey, informants who have traditional knowledge on aromatic Uyghur medicinal plants were interviewed between March 2014 and September 2014. Aromatic medicinal plant species and pertinent information were collected. Some therapeutic methods and modes of preparation of traditional aromatic medicinal plants were found.

**Results:** A total of 86 aromatic medicinal plant species belonging to 36 families were included in our study. We identified 34 plant species introduced from different regions such as Europe, India and Mediterranean areas. Fruits and whole plants were the most commonly used parts of plant, and most aromatic medicinal plants could be applied as medicine and food. We assigned the medicinal plants a use value (UV). Knowing the UV of species is useful in determining the use reliability and pharmacological features of related plants.

**Conclusions:** Xinjiang is an area in which indigenous aromatic medicinal plants are diversely used and has therefore established a sound dimensional medical healthcare treatment system. Some aromatic Uyghur medicinal plants are on the verge of extinction. Hence, further strategies for the conservation of these aromatic medicinal plants should be prioritized.

## Introduction

China is a unified multi-ethnic country, where ethnic medicine is the official unified name for the traditional medicines of Chinese ethnic minorities because of the barriers produced by the different medical systems, language, culture and species characteristics. Research based on ethnic medicinal resources is rare (Li et al. 2006). In some ethnic minority areas, the production technology of traditional ethnic medicine and clinically common and key ethnic medicinal prescriptions is facing the risk of severe loss, without being passed on to the next generation (Vandebroek & Balick 2012).

Uyghur medicine is the scientific summary and the synthesis of the wisdom of the Uyghur people, who have been hard-working in the long-term practice of production to fight diseases. Therefore, Uyghur medicine has a complete theoretical system, involving rich practical experience and a unique method of diagnosis and treatment, representing a treasure among Chinese traditional medicine.

Uyghur medicine originated from Hetian, located in Xinjiang, and has a long history (Jiang & Nie 2015). There are more than 1000 Uyghur medicinal plants on record, among which, approximately 450 are most commonly used. Most Uyghur medicines are made from plants. The Uyghur people are skilled at using aromatic drugs, which commonly involve roses, lavender (Gonçalves & Romano 2013; Mendoza et al. 2014), lip vanilla, safflower, coriander, chicory, clove (Dalai et al. 2014), cardamom (Bajaj et al. 1993) and long pepper (Tian et al. 2012; Ding et al. 2014). An aromatic plant is a plant that contains a high content of aromatic substances (essential oils or resin) that can be used as a medicine or spice. These plants are both highly useful and of high value. The aromatic medicinal species included in this report were selected according to two books, on Chinese Aromatic Plants and Uyghur Medicine. There are many aromatic plants included in records on processing and utilization in the ancient literature of China. People have often used aromatic plants for flavouring, healthcare, in wine and cosmetics, as moth repellents and refreshing substances, and for cleaning air.

Uyghur medicine is the object of this article, therefore, herbal monographs from the literature, research data, standards and regulations and physical specimens were collected, mainly to obtain information about aromatic plant varieties (Shang et al. 2012). Information about the species used in Uyghur medicines and their distribution, clinical efficacy and applied resources (preparations) was collected and reorganized, supporting the analysis, application (Auerbach et al. 2012), sharing, use and protection of Uyghur herbal resources (Zheng et al. 2006; Fred-Jaiyesimi et al. 2015).

## Materials and methods

### Study area

**Figure 1.**
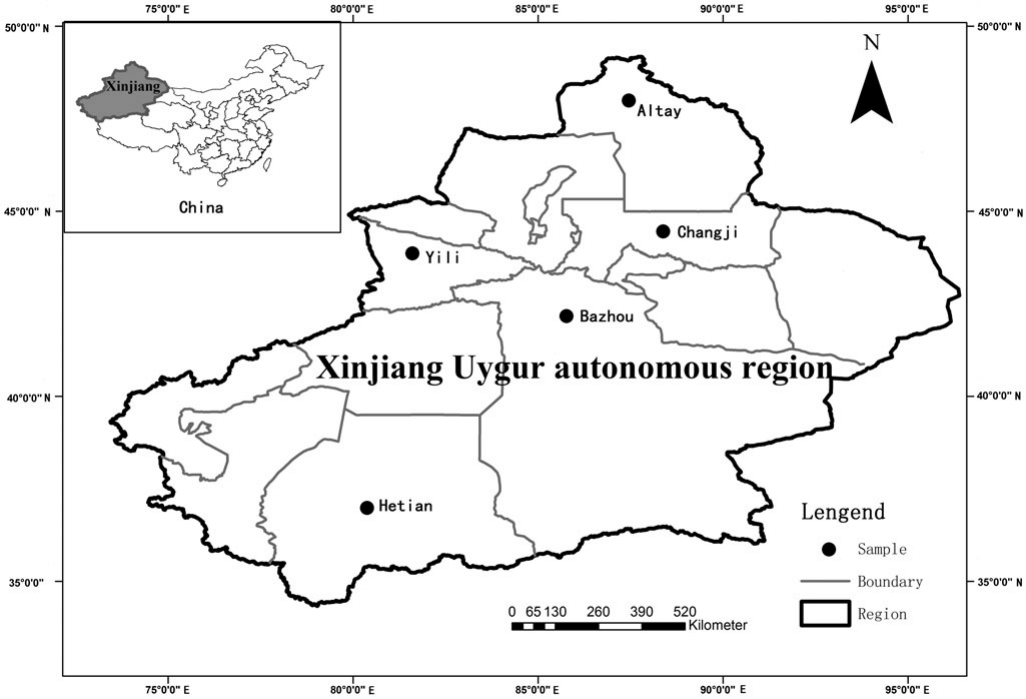
Map of the study area, Xinjiang, China.

Xinjiang Uyghur autonomous region lies in the northwest of China and is located in the centre of Eurasia. Its area is 166 km^2^, which covers 1/6 of the total area of China. Xinjiang Uyghur autonomous region has a population of more than 16.9 million, of which more than 7.906 million are Uyghur nationality. An obvious feature of the terrain is the ‘three mountain clip two basins’ (Liu et al. 2014) (Figure 1). Xinjiang is characterized by its dry climate, with the main features of sufficient sunshine and deficient rainfall. The area is far from the ocean and is surrounded by mountains, which is not only reflected in reduced moisture in the area, but also in the difference in the rainfall distribution. The Tianshan Mountains prevent cold air from flowing to the south, thus, constituting the climate demarcation line that separates the temperate zone in the north from the warm temperate zone in the south. The annual average temperature in southern Xinjiang ranges from 10 to 13 °C, whereas it is below 10 °C in the north. The average rainfall is only 45 mm, and rainfall in the north is much greater than in the south (YIN et al. 2011). Another characteristic of the area is the great discrepancy of temperature between day and night; generally, the temperature increases rapidly during the day, whereas it drops at night. People in northern Xinjiang are vulnerable to rheumatism because of the cold weather, whereas people in the south commonly suffer from liver disease, gastrointestinal disease, cardiovascular disease (Cámara-Leret et al. 2014), vitiligo and psoriasis, which can be attributed to their eating habits (giving priority to meat) and its dryness and temperature range.

The food consumed in Xinjiang is quite rich, the cooking material mainly contains meat (mutton, beef and horsemeat), dairy products and cooked wheaten food. The method of cooking them is based on roasting, stewing, steaming and so forth. For example, the popular ethnic food in Xinjiang includes mutton kebabs, kao quanyang, zhuafan, nang, etc. The people in Xinjiang basically eat meaty food. Moreover, people eating meat and grilled food are more susceptible to chronic disease. Additionally, women in a dry climate are more likely to develop various sorts of gynaecopathy. Uyghur medicine has gradually developed in areas where the above diseases have frequently occurred for quite some time.

### Field interview methods

We carried out semi-structured ethnobotanical interviews with individual natives residing in the study area in the Uyghur region between March 2014 and September 2014. A total of 200 individuals (101 men, 99 women) were interviewed in five districts, including Altay, Changji, Yili, Bazhou and Hetian. In each district, we interviewed four counties. Bazhou, Hetian are relatively large area in the south of Xinjiang. The areas are multi-ethnic areas; therefore the research on the ethnic medicine has certain representative, Altay and Yili, in the north and northwest of Xinjiang, respectively. The main nationality are Uyghur and Kazak, they have a certain understanding of the research of the ethnic medicine. Changji in the east of Xinjiang, it can be representative of the people in the east of Xinjiang on the ethnic medicine research. These five areas in Xinjiang are very representative of the region.

Interviews were conducted in bazaars, houses and parks. We confirm that the field studies did not involve endangered or protected species. Additionally, no specific permissions were required for these locations because all of the locations were public, not private. After explaining the objective of our study, we asked detailed questions related to the medicinal uses of plants (Wang et al. 2013). People who demonstrated knowledge of plants were interviewed at least twice (Polat et al. 2013). The obtained information was compared with other areas and local counties to verify its accuracy. The interviewees ranged in age from 35 to 95 years, most of whom were elders. We transcribed all interviews and deposited the recordings with the Medicinal Resources Census Project Team of China (Chen et al. 2014).

The participants provided their verbal informed consent to participate in this study. During the survey, after explaining the objective of our study, the interviewees provided us with detailed answers to questions related to the medicinal uses of plants. We subsequently transcribed all the interviews and deposited the recordings in our storehouse. All the information on aromatic Uyghur medicinal plants was recorded in tables produced by the Resource Census Project Team of China. Written consent was collected and analyzed by the authors, and the authors used another method to express the main meaning of the participants’ consent. Therefore, all of the written consents are listed in Table 1. Of course, the Medical Ethics Committees of Xinjiang Medical University approved this consent procedure.

**Table 1.** Plant species used for medicinal purposes in Xinjiang, China.

Family	Latin names	Local names	Parts used	Main chemicalcomposition ofvolatile oil	Administration form	Traditionaltherapeutic indications	The adversereactions and remedy	Way ofadministration	Use report	UV
Acoraceae	*Acorus calamus* L.	Yi ge er	Rhizome	*cis*-Methylisoeugenol, acoragermacrone, isocalamendiol, calamene	Pi, HP, Po	Sedation, anti-hypertension, anti-asthmatic, anti-tussive, spasmolysis, anti-bacterial (Vohora et al. 1990)	Harmful to brain Remedy: fennel	OR, EX	3	0.03
Amaryllidaceae	*Allium cepa* L.	Pi ya zi	The whole plant	Thiol, methyldisulphide allyl disulphide, trisulphide	Po, HP, Pou	Atherosclerosis, esoenteritis, diuretic, anti-diabetic, vitamin C supplement (Lata et al. 1991)	Harmful to brain, reduce the ability of memory Remedy: grape vinegar, honey, salt, pomegranate juice	OR, EX	1	0.01
Apiaceae	*Anethumgraveolens* L.	Se ri ke qi qie ke ou ru he	Seed	Carvone, limonene, dillapiole	D,MO	Diuretic, anti-asthmatic, anti-tussive, anti-bacterial (Tian et al. 2012)	It can reduce the ability of brain and visual acuity Remedy: sour food	OR, EX	1	0.01
Apiaceae	*Coriandrumsativum* L.	You mi ha ke su ti	The whole plant, fruit	Caparinaldehyde, nonanlan, linalool, geraniol	D, S, Pou	Clearing heat for detumescence, anti-pyrotic, diuretic (Yildiz 2016)	Excessive oral can reduced semen, and harmful to paralyzed and neurasthenia patients Remedy: honey, vitelline, long pepper and cinnamon	OR, EX	0	0.00
Apiaceae	*Cuminumcyminum* L.	Zi re	Fruit, seed	Cuminaldehyde, cuminylalcohol, α,β-phellandrene	Po, Pou, HP	Carminative, stimulate nerves, anti-bacterial, promoting digestion (Ladan Moghadam 2016)	Harmful to lungs Remedy: tragacanth gum	OR, EX	1	0.01
Apiaceae	*Daucus carota* L.	Sai wei zi ou ru he	Seed, fruit	1-limonene, cineole, geraniol, citronellol, citral, caryophyllen	Po, D,S	Inducing diuresis for treating strangurtia, dispelling cold, regulate menstrual (Rokbeni et al. 2013)	/	OR	1	0.01
Apiaceae	*Ferulaassafoetida* L.	Ying ou ru he	Seed	(r)-2-Buty-1-propenyl, disulphide, α-pinene, phelladrine	Po, HP, Pou	Dispel the wind, relieve pain, enhance memory, diminish inflammation, apocatastasis (Zia-Ul-Haq et al. 2012)	Harmful to intestinal disease and cystipathy patients Remedy: semen melo, acacia	OR, EX	0	0.00
Apiaceae	*Ferula.sinkiangensis*K. M. Shen.	Ying	Resin	α-Pinene, phellandrine, α-terpineol, bornyl acetate	Pi, Po, HP	Arthralgia, paralysis, traumatic injury (Li et al. 2011)	Forbidden for pregnant women, harmful to brain and liver Remedy: acacia, anise, pomegranate fruit	OR, EX	0	0.00
Apiaceae	*Foeniculum vulgare* Mill.	A ri pa ba di yang	Fruit, root bark	*trans*-Anethole, fenechone, linoene, β-pinene, methyl chavicol	HP,S,D	Anti-tumour, cholagogue, inhibition of gastric ulcer, anti-bacterial (Rahimi & Ardekani 2013)	Harmful to febrile healthy Remedy: sandalwood	OR, EX	10	0.11
Apiaceae	*Pimpinella anisum* L.	Ru mi bie di yang	Seed	Anisaldahyde, amisic acid, anethele, anisyloectone	HP, S, Pi	Facial paralysis, headache, amenorrhea, exhausting qi, prolactin (Samojlik et al. 2012)	Harmful to intestinal disease Remedy: fennel	OR, EX	1	0.01
Apiaceae	*Pleurospermum lindleyanum* (Lipsky) B. Fedtsch.	Yu re ke ou ti	The whole plant	α-Pinene, myristicin, elemicin, asarone, ocimene phellandrine	A,MT,S	Coronary, heart disease, anaesthesia, anti-asthmatic, anti-tussive, anti-hypertension	/	OR	0	0.00
Apiaceae	*Ferula. fukanensis* K. M. Shen.	Ying yi li mi	Resin	(R)-2-Buty-1-propenyl, disulphide, α-pinene, phelladrine, undecylsulfony acetic acid	Pi, HP, Po	Anti-anaphylaxis, anti-inflammatory, arthralgia, traumatic injury, paralysis (Sahebkar et al. 2011)	Forbidden for pregnant women, harmful to brain and liver Remedy: acacia, anise, pomegranate fruit	OR, EX	1	0.01
Apocynaceae	*Nerium indicum* Mill.	Su gai ti gu li	Leaves, bark, root	Menthyl salicylate, acroleic acid, butanone alcohol, ethyl sulphide, ethyl acetate	MO, Pou, O	Cardiotonic action, diuretic, sedation (Dey & Chaudhuri 2013)	Rank poison, harmful to brain and lungs, can make people dazzled remedy: milk, grease	EX	0	0.00
Arecaceae	*Areca catechu* L.	Fu pai li	Seed	/	Po, D, HP	Insect repellent, against pathogen, increase appetite, anti-cancer, anti-hypertensive, antioxidant (Phaechamud et al. 2009)	Lead to chest and lung dryness, kidney stone and vesical calculus remedy: tragacanth gum	OR, EX	0	0.00
Arecaceae	*Cocos nucifera* L.	Na er ji li	Fruit	2-Heptanone, 2-nonanone, dodecylic acid, n-amyl butyrate, γ-decanolactone	HP, Po	Tonifying brain, psychosis, hypochondria, tocolysis (Lima et al. 2015)	It cannot be digested easily Remedy: sugar candy, fresh fruit	OR, EX	2	0.02
Araliaceae	*Panax ginseng* C.A.Mey.	A dai mu ge ya	Root	Panaxynol, elemene β-aromadendrene, tetradecanoic acid, cetylic acid	D, HP	Neurasthenia, amnesia, vasodilation, anti-shock, anti-hypertension, promoting metabolism (Ru et al. 2015)	It cannot be eaten with helleborus thibetanus Remedy:/	OR	0	0.00
Araliaceae	*Panax notoginseng* (Burk.) F. H. Chen	San qi	Root	Spathulenol, heptane, γ-sitosterol, panaxynol, ethyl linolenate	D, Po, Pi	Dilate the coronary arteriae, increased coronary flow, resisting acute myocardial, ischaemic injury, anti-hypertension, haematolysis, anti-inflammatory (Wang et al. 2016)	/	OR, EX	0	0.00
Aristolochiaceae	*Asarumeuropaeum* L.	A sa rong	The whole plant	Asarone, d-asarone asarylaldehyde,1-pinene, eugenol, methyleugenol, bomylacetate	HP, D, Po	Local anaesthesia, anti-pyretic analgesic, anti-bacterial, anti-hypertension (Sadati et al. 2016)	Harmful to liver Remedy: raisin grape or sophora flower	OR, EX	1	0.01
Brassicaceae	*Brassica juncea* (L.) Czernet Coss	Ke zi li ke zha	Seed	Dolcymene, methyl-isorhodanate, butyl isothiocyanate, propyl isorhodanate, benzene methyl, isocyanate	Pou	Anti-bacterial, increase appetite, improve blood circulation, expectorant emetic	/	OR, EX	0	0.00
Brassicaceae	*Sinapis alba* L.	A ke ke zha	Seed	/	Pou		Excessive oral can make people thirsty easily Remedy: chicory grape vinegar	OR, EX	2	0.02
Burseraceae	*Boswellia carterii* Birdw	Kun du er	Resin	Pinene, dipentene, α,β-phellandrene	HP, Po, Pou	Anti-Inflammatory, enhance memory, bronchiectasia, acesodyne, cacochylia (Prakash et al. 2014)	Excessive oral can cause headache Remedy: granulated sugar	OR, EX	0	0.00
Compositae	*Artemisiaabsinthium* L.	A qi ke ai man	Leaves	Thujone, thujol	S, D, T	Iaryngopharyngitis, amygdalitis typhoid, fever, hepatitis, pericarditis eczema (Rajesh Kumar 2013)	Excessive oral can cause headache Remedy: anisum	OR, EX	3	0.03
Compositae	*Artemisia argyi* Lévl.et Vant	Ai man	Leaves	Phellandrene, cadinene, thujyl alcohol	D, S, Pou	Anti-bacterial arthralgia, oedema, dystocia, oligotrichosis (Wenqiang et al. 2006)	Excessive oral can cause headache, harmful to kidney Remedy: anisum, mastiche	OR, EX	0	0.00
Compositae	*Artemisia rupestris* L.	Yi zi qiu ai mi ni	The whole plant	Linallol, p-cymene, α-terpineol, β-pinene terpinen-4-ol, α-pinene	D, S, Pi	Anti-anaphylaxis, cold, fever, headache, stomach ache, hepatitis (Ji et al. 2007)	/	OR,EX	0	0.00
Compositae	*Aucklandia lappa* Dence.	Ku si tai	Root	β-Elemene, globulol, α-muurolene, dehydrocostus lactone, costunolide	HP, S, Po	Appetizing, acesodyne, insect repellent, aphrodisiac, hepatalgia pneumalgia, anti-allergic (Seo et al. 2015)	Harmful to bladder and lungs Remedy: anisum, flos rosae rugosae, massecuite	OR, EX	4	0.05
Compositae	*Carthamustinctorius* L.	zha rang za qie qi ke	Flower	Decahydrate,3,3-dimethyl-heptane, 2,2,4-trimethyl-3-amylketone, octane	D, HP, S	Irregular menstrual, impotence, asthma, leucoderma, eczema (Zhang et al. 2016)	Excessive oral can cause headache, harmful to gastrosis and throat disease Remedy: anisum and honey	OR, EX	0	0.00
Compositae	*Cichorium intybus* L.	Ka si ni	The whole plant	Cichoriin, diacetyl, furaldehyde, furan, lactate, maltol	S, D	Hepatitis, gastritis, jaundice, splenauxe, oedema (Li et al. 2015)	Excessive oral can cause cough Remedy: white sugar or viola tianshanica maxim	OR, EX	7	0.08
Compositae	*Dendranthemamorifolium* (Ramat.) Tzvel.	Ju hua gu li	Capitulum	d-verbenol, bomyl acetate, cinenle, (—)-zingibenene, cuhenol, α-fmesene	D, S	Disintoxication,anti-pruritic, liver heat, ophthalmodynia, detumescence, anti-bacterial (Wang et al. 2013)	Harmful to cold property of body Remedy: fennel	OR	0	0.00
Cucurbitaceae	*Cucumis melo* L.	Kuo hun	Fruit, seed, pedicel	Ethyl acetate,2-methylbutanol,2-phenethyl alcohol, 2-methyl-1-propanol,1-heptanol	D,S	Dry stool, dysuria, emaciation, stomach discomfort, quench one's thirst	Excessive oral can cause diarrhoea, fever and various of eye diseases Remedy: pomegranate juice, honey, mastic, rhizoma zingiberis	OR	0	0.00
Cucurbitaceae	*Cucurbitamoschata* L.	Kuo ke ka wa ou ru he	Seed	Linoleic acid, oleic acid, palmitic acid, stearic acid, linolenic acid, myritic acid	Po, cataplasm	Fever, oedema, acute pneumonia, insect repellent, anti-schistosoma	Harmful to cold property of body Remedy: fennel, black pepper	OR	0	0.00
Cupressaceae	*Sabina vulgaris* Antoine	A ri cha mei wei si	Cone	Sugiol, deoxypodophyllotoxin,sabina coumarin, β-sitosterol, myristic acid lactone	D, S, T, Po	Amenorrhoea,stomach cold, abscess, abscess, black shading, gingival erosion (Wu-Bao et al. 2005)	Harmful to lungs remedy: tragacanth gum cannot be used by pregnant woman, gastrosis and throat disease Remedy: galangal, honey	OR, EX	0	0.00
Elaeagnaceae	*Elaeagnusangustifolia* L.	Ji ge de qie qi ke	Flower	Phenethyl alcohol, methyl cinnamate, palmitic acid, ethyl palmitate, ethyl oleate, nonadecanoic acid	S, Pou	Inhibited sexual desire, asthma, pectoralgia, prevent disease (Farzaei et al. 2015)	Young girl and unmarried young woman had not to smell Remedy:/	OR, EX	0	0.00
Elaeagnaceae	*Elaeagnus rhamnoides* L. subsp.*sinensis* Rousi	Ji hang	Fruit	n-Tetradecanal,n-pentadecanal,1,1-diethoxy-n-nonane, α,β-lonone,1,1-diethoxy-n-tetradecane	S,D,	Anti-tussive, anti-emetic, relieving asthma, anti-hypertension, cacochylia, increase vitamin (Tolkachev et al. 2008)	/	OR	0	0.00
Ephedraceae	*Ephedra equisetina* Bunge	Zha kang da	Herbaceous stem, root	4-Terpineol, butylated hydroxytoluene, patchoulene, octyl pyridazine ring, (4αR-t)naphthalene	D, S, Po	Cough, relieving asthma, cold, pneumonia, night sweat, diarrhoea, skin and external diseases (Yoshizawa et al. 2004)	Deleteriousness Remedy:/	OR, EX	0	0.00
Euphorbiaceae	*Ricinus communis* L.	Yi nai ke pi ti ou ru he	Seed	Ricinolic acid, glyceride, isopropylricinoleic acid, palmitic acid, octadecanoic acid	Pi, HP, Pou	Facial paralysis, arthritis, cough, headache, celiodynia, astriction, cerebral haemorrhage (Zarai et al. 2012)	Deleteriousness, excessive oral can decreased digestive function, cause vexation, naupathia, vomit Remedy: tragacanth gum, mastic, ageratum	OR, EX	0	0.00
Gentianaceae	*Gentiana scabra* Bunge	Jin ti ya na	Root, rhizome	Methyl benzenecarboxylate, 1-octadecene, 1-hexadecene,9-eicosylene,3-nitro-1,2-m-phthalic acid	D, Pi, Po	Cacochylia, detoxification, detumescence, acesodyne, paralysis, rabies (Huang et al. 2014)	Harmful to hot property of chest of body Remedy: centipede	OR, EX	1	0.01
Iridaceae	*Crocus sativus* L.	Zai fa er	Stigma	Palmitic acid, palmitoleic acid, oleic acid, linoletic acid, lindenic acid, β-sitosterol	HP, S, D	Dismayed, insomnia, congestion, amenorrhea, anti-hypertension, heart disease, hysteroptosia, vitiligo (Ayatollahi et al. 2014)	Harmful to kidney, can cause inappetence Remedy: anisum, vinegar syrup, amur corktree	OR, EX	21	0.24
Lamiaceae	*Agastache rugosa* (Fisch. et Mey.) O. Kuntze	Pin nai	Acrial part	Methylchavicol,anethole, anisaldehyde, patchoulialcohol, α,β-pinene,d-limonene	Po, flowerpaste, S	Neurasthenia, gastrointestinal disease, anti-hypertension, anemofrigid headache, toothache, earache (Dũng et al. 1996)	Excessive oral can cause ventosity, dry throat Remedy: celery	OR, EX	2	0.02
Lamiaceae	*Dracocephalummoldavica* L.	Ba de ran ji bu ya qi ne	The whole plant	Citral, geraniol, nerol, citronellol, thymol	D, S, lotion	Heart disease, vexation, dizziness, cough asthma, detoxification, halitosis (Maimaitiyiming et al. 2014)	/	OR, EX	7	0.08
Lamiaceae	*Lavandula angustifolia* Mill.	Wu si tu hu du si	Aerial part	Geraniol, safrole,carvacrol, linalool, citionellor	Essential oil	Nervous system disease, paralysis, amnesia, melancholia, arthralgia (Mendoza et al. 2014)	Harmful to hot property of body Remedy: acetic acid syrup	OR	7	0.08
Lamiaceae	*Melissa officinalis* L.	Ba de ran ji bu ya qi ni	The whole plant	Citral, ctronellal, geraniol, linalool	S, Pou, A	Sterilization, stenocardia, anti-hypertension, anti-sepsis insect repellent (Shakeri et al. 2016)	Excessive oral can cause ribs pain Remedy: gummi arabicum, mastic	OR, EX	0	0.00
Lamiaceae	*Mentha canadensis* L.	Ya li pu zi	The whole plant, leaves	Methol, menthone,thymol, carvacrol, β-eugenic acid,p-cymene	HP, S, Pou	Amenorrhoea, difficult urination, abdominal pain, expectorant, anti-bacterial, anti-inflammatory, anti-viral (Jirovetz et al. 2009)	Harmful to anus Remedy: gummi arabicum, grape vinegar	OR, EX	4	0.05
Lamiaceae	*Ocimum album* L.	Ya wa re yi han	The whole plant	Caryophyllic acid, eugenol, caryophyllene, methyleugeno	D, S, Po, MO	Heart deficiency, palpitation, impotence, amenorrhoea, gastric asthenia (Dhima et al. 2010)	Excessive oral can cause dizzy remedy: grape vinegar, cucumber, purslane	OR, EX	0	0.00
Lamiaceae	*Ocimum basilicum* L.	Re yi han	Aerial part, seed	Ocimene, α-pinene,1,8-cineole, linalool, geraniol, methyl cinnamate	D, S, Pou	Hepatopathy, cardiopalmus, melancholia, paralysis, arthralgia, diarrhoea (Govindarajan et al. 2013)	Harmful to eye, can reduce vision Remedy: grape vinegar or purslane	OR, EX	1	0.01
Lamiaceae	*Ocimum gratissimum* L. var. suave (Willd.) Hook. f.	Pai ran ji mu xi ke	The whole plant	3-Haxen-1-ol, thujene, sabinene, α-humulene, β-cubebene, copaene	D, S	Liver vacuity, palpitation, cold, antibechic, gastric asthenia expectorant (do Nascimento Silva et al. 2016)	Excessive oral can cause headache, stomach acid reflux remedy: grape vinegar, viola tianshanica maxim	OR, EX	2	0.02
Lamiaceae	*Origanummajorana* L.	Mai er zan zhu xi	The whole plant	Thymol, carvacrol, geranyl acetate, α,β-pinene, linalool	D,S	Cold headache, palpitation, peripheral facial paralysis, intestinal obstruction (Hajlaoui et al. 2016)	Harmful to kidney bladder Remedy: purslane, chicory	OR	0	0.00
Lamiaceae	*Perilla frutescens* (L.) Britt. var. acuta (Thunb.) Kudo	Ba lan gu	The whole plant	Perillaldehyde, elsholtzia alcohol, menthanol, eugeno, linalool, olivil	D, Po	Heart deficiency, palpitation, vomitus gravidarum, threatened abortion, headache, chest tightness (Chen et al. 2004)	Harmful to stomach Remedy: white crystal sugar	OR	1	0.01
Lamiaceae	*Thymus vulgaris* L.	A sha	The whole plant	Linalyl acetate, bornyl acetate, caryophyllene, thujanol-4, terpineol-4, borneol	Pou, embrocation	Liver vacuity, gastric asthenia, anuresis, amenorrhoea, facial paralysis, asthma, haemoptysis (Youdim & Deans 1999)	Harmful to pneamopathy Remedy: concretio silicea bambusae, ageratum	OR, EX	0	0.00
Lamiaceae	*Ziziphora clinopodioides* Lam.	Su ze	The whole plant	α,β-Pinene, pulegone, β-citronellol, β-caryophyllen, ylangene	D, MT, lotion	Cold, fever, headache, palpitation, insomnia, oedema, sore throat, rickets, asynodia (Tian et al. 2012)	/	OR, EX	0	0.00
Lauraceae	*Cinnamomum cassia* Presl	Da er qin	Dried bark	Cinnamaldehyde, cinnamyl acetate, anisaldehyde, t-cinnamaldehyde, benzaldehyde, salicylaldehyde	D, HP, Pou	Stomach cold, diarrhoea, dyspepsia, palpitation, ventosity, hepatic asthenia, asynodia (Ooi et al. 2006)	Harmful to bladder Remedy: tragacanth gum or asarum europaeum	OR, EX	19	0.22
Leguminosae	*Dalbergia odorifera* T. Chen	Jiang xiang	Trunk, heartwood	β-Bisalolene, (E)-β-farnesen, (E)-nerolidol, nerolidol	Pi, HP, A	Traumatic injury, neurasthenia, vexation, thermalgia, pyogenic infections (Chan et al. 1998)	/	OR, EX	0	0.00
Leguminosae	*Glycyrrhiza uralensis* Fisch.	Qu qu ke bu ya	Root, rhizome	Nonadecane, larane, octadecane, (E)-pinane, cetyl-epoxyethane, docosane	T, D, S	Anaudia, asthma, cough, lung diseases, cold and fever detoxification, anti-tumour, antioxidant (Gong et al. 2015)	Harmful to kidney and spleen Remedy: tragacanth gum and flos rosae rugosae	OR, EX	13	0.15
Leguminosae	*Trigonella foenum-graecum* L.	Shu mi sha ou ru he	Seed	Hexanol, heptanone, enanthal, cineole, thymol, camphor	D, Po, HP	Lymphatic tuberculosis, hoarseness, amenorrhoea, hyposexuality, herpes (Goyal et al. 2016)	Harmful to hot property of body, can cause headache Remedy: spinach and purslane Harmful to testicle Remedy: costus oil Excessive oral can cause nausea and vomit remedy: vinegar syrup, anisum	OR, EX	1	0.01
Moraceae	*Ficus carica* L.	An ji er	Receptacle of inflorescence	Furfural, phenylacetaldehyde, 2-acetylpyrrole, ethyl linoleate, linolenic acid, phytol	S,D	Cough, inappetence, constipation, infantile paralysis, irregular menses, cacotrophy (Harzallah et al. 2016)	Excessive oral is harmful to hepatic asthenia and gastric asthenia patients Remedy: walnut, anisum	OR	1	0.01
Myristicaceae	*Myristica. fragrans* Houtt.	Zhu you zi	seed	Sabinene, α,β-pinene, terpinen-4-ol, limonene, bornylene, β-phellandrene	HP, S, Pou	Dyspepsia, arthritis, cold headache, pyocutaneous, asynodia, diarrhoea (Bajaj et al. 1993)	Harmful to hot property of body Remedy: eat with coriander Harmful to liver and lungs Remedy: eat with honey and viola tianshanica maxim Excessive oral can cause aphonia and hoarseness Remedy:/	OR, EX	15	0.17
Myrtaceae	*Myrtus communis* L.	Ai bu li a si	Fruit	Pinene, camphene, cineole, cinene, geraniol	S, HP, Po	Gingival bleeding, haematuria, diarrhoea, hypermenorrhoea, abscess, trichomadesis (Ebrahimabadi et al. 2016)	Harmful to brain, can cause headache, insomnia Remedy: viola tianshanica maxim	OR, EX	2	0.02
Myrtaceae	*Syzygium aromaticum* (L.) Merr. et Perry	kai lan fu er	Flower bud	Eugenol, acetyleugenol, humulene, β-caryophyllene	Po, D, HP	Gastric asthenia, dyspepsia, arthritis, paralysis, amnesia (Dalai et al. 2014)	Harmful to hot property of body, kidney, and intestines Remedy: gummi arabicum	OR	11	0.13
Nymphaeaceae	*Nymphaea candida* Presl	Ni lu fa er	Flower	/	D, S, lotion	Heart deficiency, liver vacuity, cough, cold, vexation, thirsty, anti-hypertension	Harmful to bladder Remedy: honey, crystal sugar	OR, EX	4	0.05
Papaveraceae	*Papaver somniferum* L.	kuo ke na er po si ti	Shell	2,4-Nonadienal, 2,4-decene aldehyde, cyclopentadecane, hexanal, docosane	D, Po, S	Cough, insomnia, cephalagra, haematemesis, hemafecia, kidney deficiency, diarrhoea (Paul et al. 1996)	Harmful to brain and pneamopathy patients Remedy: honey, fennel, granulated sugar, mastic	OR	17	0.20
Piperaceae	*Piper nigrum* L.	Mu qi	Fruit	Piperonal, dihydrocarveol, caryopyllene oxide, cryptone, phellandrene, cis-p-2,8-menthadienol	HP, Pou, Po	Dyspepsia, abdominal distension, cough, headache, toothache, anti-inflammatory (Bagheri et al. 2014)	Harmful to hot property of body, can cause headache, dryness of the throat and lungs Remedy: cold property of oil	OR, EX	14	0.16
Ranunculaceae	*Nigella glandulifera* Freyn et Sint.	Si ya dan	Seed	Thymoquinone, nigellon	HP, Po, injection	Vitiligo, amnesia, tremor, ventosity, bellyache, amenorrhoea, oedema (Ghanemi & Boubertakh 2015)	Harmful to hot property of body, Remedy: eat after soaking in the grape vinegar Harmful to kidney remedy: tragacanth gum	OR, EX	7	0.08
Ranunculaceae	*Paeonia lactiflora* Pall.	Ke zi li chu hu lu ke	Root tuber	β-Phenylethyl alcohol, citronellol, hexenoic aldehyde	HP, Pi, Po	Epilepsy, paralysis, psychosis, phobia, encephalitis, irregular menses (Wang et al. 2014)	Harmful to pregnant woman Remedy: nectar	OR, EX	0	0.00
Rosaceae	*Agrimonia eupatoria* L.	Ha pai si	The whole plant	3-Hydroxybutyric acid, α-bisabolol, ledol, tetratriacontane, 2,6-di-tert-butylphenol	D, Po, Pou	Chronic hepatitis, oedema, urination, eczema, alopecia areata (Muruzović et al. 2016)	Harmful to spleen and testicle Remedy: anisum	OR, EX	0	0.00
Rosaceae	*Crataegus pinnatifida* Bunge	Du la nai	Fruit	Maslinic acid, palmitic acid, octadecanoic acid, linolenic acid, chlorogenic acid	D, Po, S	Gastrectasia, dyspepsia, diarrhoea, dysentery, hepatic asthenia, hyperlipidaemia, (Li et al. 2010)	Harmful to kidney, gastric asthenia and enteropathy patients, can cause headache, bowel infarction Remedy: anisum, fennel, ligaloes, qizil guliqent	OR, EX	0	0.00
Rosaceae	*Eriobotrya japonica* (Thunb.) Lindl.	Luo ka ti	Fruit	Nerolidol, farnesol, camphene, p-cymene, linalool, myrcene	S,D	Fever, retch, vexation, thirsty (Ge et al. 2009)	Excessive oral can cause cough Remedy: hot property of food	OR	0	0.00
Rosaceae	*Malus pumila* Mill.	A li ma	Fruit	Farnesene, malic acid, ethyl salicylate, ethyl lactate, citronellol	S	Inappetence, constipation, diarrhoea, hepatic asthenia, gastric asthenia (Bai et al. 2016)	Excessive oral can cause typhoid, amnesia, pneumatosis, muscle spasm Remedy: honey, cinnamon	OR, EX	2	0.02
Rosaceae	*Prunus armeniaca* L.	Ou ru ke	Fruit	Terpinenol-4, linalyl formate, ethyl myristate, γ-caprylolactone, isobutyric acid	S, infusion	Dry stool, fever, stomach heat, haemorrhoids, thirsty (Lee et al. 2014)	Harmful to elderly persons and gastritis of insufficiency-cold patients Remedy: granulated sugar, anisum, ajowan-caraway seed	OR	0	0.00
Rosaceae	*Prunus domestica* L.	Ai nu la	Fruit	/	Extract, D,S	Fever, typhoid fever, pulmonary tuberculosis, tussiculation, acute laryngopharyngitis, diarrhoea, vitamin C deficiency (Rahim et al. 2015)	Harmful to cold property of body, harmful to brain, gastrosis patients remedy: mastic, honey water	OR	0	0.00
Rosaceae	*Prunus persica* (L.) Batsch	Sha pi tuo li	Fruit	Malic acid, citric acid	D, Jam agent, MO	Dry stool, typhoid fever, gastric asthenia, hepatic asthenia, thirsty (Han et al. 2015)	Harmful to cold property of body Remedy: honey	OR, EX	0	0.00
Rosaceae	*Pyrus sinkiangensis* Yu	Nai xi pu ti	Fruit	Ethyl butyrate, ethyl caproate, hexanol, ethyl palmitate, α-farnesene	S,D	Stomach heat, thirsty, coprostasis, weak health	Harmful to cold property of body and gastric asthenia patients Remedy: fennel, ginger	OR	0	0.00
Rosaceae	*Rosa chinensis* Jacq.	Ai ti ri gu li	Flower bud	Geraniol, nerol, citronellol, coriandrol	Flower paste	Neurasthenia, gloomy, amenorrhoea, anti-bacterial (Pei et al. 2014)	Harmful to hot property of body Remedy: basil flower	OR	0	0.00
Rosaceae	*Rosa rugosa* Thunb.	ke zi li gu li	Petal	Linabool, linalyl formate, β-citronellol, citronellyl formate, β-damascone, roseoxide	D,T	Hepatitis, neurasthenia, palpitation and insomnia, megrim, coprostasis, myocarditis (Gonçalves et al. 2013)	Excessive oral can cause sexual function decline Remedy: anisum	OR, EX	19	0.22
Rutaceae	*Citrus limon* Burm.	Li meng	Fruit	d-limonene, citral, gerangl-acetate, linalyl-acetate	S, D, Pou	Headache, pharyngalgia, palpitation, vomit, nausea, cold, thirsty (Settanni et al. 2014)	Harmful to cold property of body Remedy: granulated sugar	OR	1	0.01
Rutaceae	*Citrus medica* L.	Tu run ji po si ti	Pericarp	Hesperidin, nobeletin, β-phellandrene, α-terpinene, inose	D, S, Po	Dyspepsia, diarrhoea, nausea, vomit, gastric asthenia, black shading (Menichini et al. 2011)	Excessive oral can cause hot property of body headache Remedy: honey, viola tianshanica maxim	OR, EX	0	0.00
Rutaceae	*Ruta graveolens* L.	Suo za bi	The whole plant	α,β-Pinene, linalool, camphorene, ρ-cymene, cineole, camphene	HP, D, Po	Dysuria, arthralgia, otalgia, convulsion, menstrual disorder, mental decline, paralysis, vitiligo (Ratheesh et al. 2011)	Harmful to eye, can cause headache Remedy: anisum	OR, EX	2	0.02
Rutaceae	*Zanthoxylum bungeanum* Maxim.	Ka ba bai qi ni	Pericarp	Limonene, cumic alcohol, geraniol, estragole, chavicol methylether	HP, Po, S	Dyspepsia, gomphiasis, ozostomia, leucorrhagia, local anaesthesia, anti-inflammatory, insect repellant (Rong et al. 2016)	Harmful to bladder Remedy: mastic can cause crotch pain Remedy: sandalwood	OR, EX	3	0.03
Schisandraceae	*Illicium verum* Hook.f.	Sha ka li ba di yang	Fruit	Anethole, methylchavicol, safrole, 1,8-cineole, aubepine, fenchone	D, Pou	Gastric asthenia, emesis, abdominal pain, lumbago due to deficiency of the kidney (Zhang et al. 2015)	Excessive oral can cause headache Remedy: long pepper	OR, EX	0	0.00
Smilacaceae	*Smilax china* L.	Qie bi qi ni	Rhizome	/	HP, Po, S, D	Headache, paralysis, melancholia, arthralgia, hepatic asthenia, ahypnosis, menostasis (Chen et al. 2011)	Harmful to hot property of body Remedy:/	OR	3	0.03
Solanaceae	*Lycium chinense* Mill.	A li ha ti	Fruit	Ionone, benzil alcohol, phenylmethyl acetate, methyl linoleate, 7,10,13-hexadecatrienoic acid methyl ester	D,S	Hypaphrodisia, spermatorrhoea, hepatic asthenia, neurosism, hyperglycaemia, hyperlipoidaemia (Olatunji et al. 2015)	Harmful to loose stool patients Remedy: fructus aristolochiae	OR	0	0.00
Styracaceae	*Styrax benzoin* Dryand.	Luo bang	Balm	Liquid storax, cinnamyl benzoate, vanillin, benzoic acid, α-cedrene	Po, Pi, O	Cold, cough, asthma, bronchiectasis, kidney calculi, pyocutaneous, haemorrhage, asynodia (Pastorova et al. 1997)	Harmful to hot property of body Remedy: viola tianshanica maxim, poppy	OR, EX	0	0.00
Thymelaeaceae	*Aquilaria agallocha* Roxb	Ou di yin di	Resin wood	α-Agarofuran, agarol, agarospirol, jinkoheremol, kusunol, didrikaranone	S, D, Po	Gastric asthenia, arthralgia,halitosis, cough, asthma, ahypnosis, (Bhuiyan et al. 2008)	Harmful to hot property of body Remedy: clove, cassia twig, saffron, dutohmanspipe fruit	OR, EX	4	0.05
Vitaceae	*Vitis vinifera* L.	Ou ru he si zi ou zu mi	Fruit	β-Myrcene, myrcene, hexenoic aldehyde, geranic acid, p-toluene	D,S,HP	Constipation, hepatic asthenia, asynodia, melancholia (Liu et al. 2012)	Harmful to hot property of body Remedy: acid fruit	OR	2	0.02
Zingiberaceae	*Amomum tsao-ko* Crevost & Lemarié	Chong ka ke le	Fruit	α,β-Pinene, α-terpineol, neral, geraniol, linalool	Po, D,HP	Stomach cold, anorexia, ventosity, diarrhoea, loose stool (Shin et al. 2016)	Harmful to lungs Remedy: cube sugar excessive oral harmful to intestinal tract Remedy: tragacanth gum	OR	7	0.08
Zingiberaceae	*Curcuma longa* L.	ze qi wai	Root	Turmerone, arturmerone, zingiberene, phellandrene, sabinene	D, HP, Pou	Traumatic injury, oedema, toothache, cineole, cough, cataract, trachoma, asthma, dermatosis (Parveen et al. 2013)	Harmful to heart Remedy: lemon juice, orange juice	OR, EX	0	0.00
Zingiberaceae	*Elettaria cardamomum* Maton	La qin da nai	Fruit	Terpineol-4, α-terpineol, terpinylacetate, cineole	D,HP	Gastric asthenia, ozostomia, dyspepsia, ventosity, vomit, nausea, bellyache, palpitate (Nigam et al. 1965)	Harmful to lungs remedy: tragacanth gum, concretio silicea bambusae	OR, EX	4	0.05
Zingiberaceae	*Zingiber officinale* Roscoe	Zan ke bi li	Rhizome	α-zingiberene, geranial, geraniol, isogingerenone, hexahydrocurrumin, 6-gingerol	Pi, Po, Pou	Gastric asthenia, anemofrigid cold leucorrhagia, asynodia, loose stool (Heeba & Abd-Elghany 2010)	Harmful to throat Remedy: honey	OR, EX	12	0.14

Administration form: S: syrup; HP: honey paste; Pou: poultice; Pi: pill; Po: powder; D: decoction; A: apozem; MO: medicinal oil; MT: medicinal tea; T: tablet.

Way of administration: OR: oral EX: external.

/No up-to-date report was there on these aspects.

**Figure 2.**
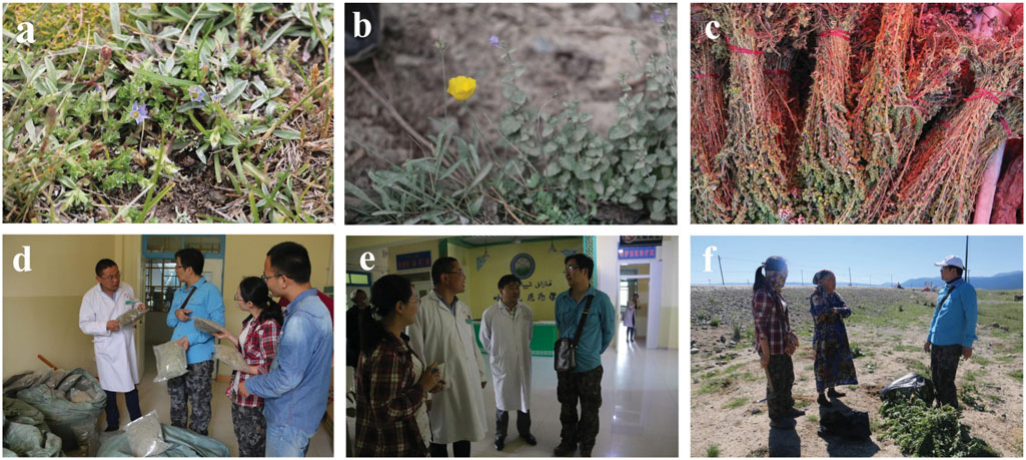
Aromatic Uyghur medicinal plants and field interview. (a) *Gentiana scabra* Bunge, (b) *Papaver somniferum* L., (c) commercially available *Artemisia rupestris* L., (d) field interview about *Ziziphora clinopodioides* Lam., (e) field interview about aromatic Uyghur medicinal plants in ethnological hospital, (f) field interview about *Melissa officinalis* L. with retired ethnological doctor.

The interview questions were aimed at understanding the traditional uses of medicinal plants, including local plant names, ailments for which the plants were used, the parts of the plants used, and methods of preparation and administration. We accompanied the interviewees into the field to collect specimens of the plants to which they were referred. We also deposited the plant materials collected in our study with the Medicinal Resources Census Project Team of China (Figure 2).

### Voucher specimen collection

To exemplify and protect the aromatic medicinal plants obtained in Xinjiang to the best extent possible, we collected voucher specimens between March and September 2014. Voucher specimens were collected and prepared under the directions of herbalists and local people, who have much experience with these aromatic Uyghur medicinal plants. The plants were identified by a research team specialized in Uyghur medicinal resources, consist of several pharmaceutical professors and several graduate students from Xinjiang Medicine University, and specimens were deposited in the Traditional Chinese Medicine Voucher Herbarium of Xinjiang Medicine University. All data were collected in a database.

### Data analysis

The use value (UV), a quantitative index that indicates the relative importance of locally known species, was also calculated according to the following formula: UV = *U*/*N*, where *U* is the number of reported uses cited by each informant for a given species, and *N* refers to the total number of reports in which UV refers to the UV of a species. UVs are high when there are many reported uses for a plant, thereby indicating that the plants are actively used by local people, whereas when there are few reports related to a plant’s use, the UV approaches zero (0) (Boakye et al. 2015). Therefore, knowing the UV of a species may be useful in determining the reliability of the use and pharmacological features of related plants.

## Results and discussion

### Families and medicinal plants

A total of 86 aromatic medicinal species belonging to 36 families were included in the present study (Table 1). About 12 medicinal species belonged to Lamiaceae, which was the family with the highest percentage (13.95%) of medicinal species used by the Uyghur people, followed by Apiaceae and Rosaceae (11.63%) with 10 species, and Compositae (9.30%) with 8 species. These four families account for 46.51% of the total number of aromatic medicinal species identified. The remaining 46 species belongs to 8 other families with less than six species each, while only one species was obtained for approximately 20 families (Table 2).

**Table 2.** Frequency of plant species by family used for medicinal purposes in the study area.

Family	Frequency	Family	Frequency
Acoraceae	1	Leguminosae	3
Amaryllidaceae	1	Gentianaceae	1
Apiaceae	10	Moraceae	1
Apocynaceae	1	Myristicaceae	1
Araliaceae	2	Myrtaceae	2
Arecaceae	2	Nymphaeaceae	1
Aristolochiaceae	1	Papaveraceae	1
Brassicaceae	2	Piperaceae	1
Burseraceae	1	Ranunculaceae	2
Compositae	7	Rosaceae	10
Cucurbitaceae	2	Rutaceae	4
Cupressaceae	1	Schisandraceae	1
Elaeagnaceae	2	Smilacaceae	1
Ephedraceae	1	Solanaceae	1
Euphorbiaceae	1	Styracaceae	1
Iridaceae	1	Thymelaeaceae	1
Lamiaceae	12	Vitaceae	1
Lauraceae	1	Zingiberaceae	4

In the analysis conducted in this study, many species collected in Xinjiang were observed to be used medicinally and were easily accessed (Liu & Shawuti 1985; Liu 1999).

### Plant parts and mode of preparation

**Figure 3.**
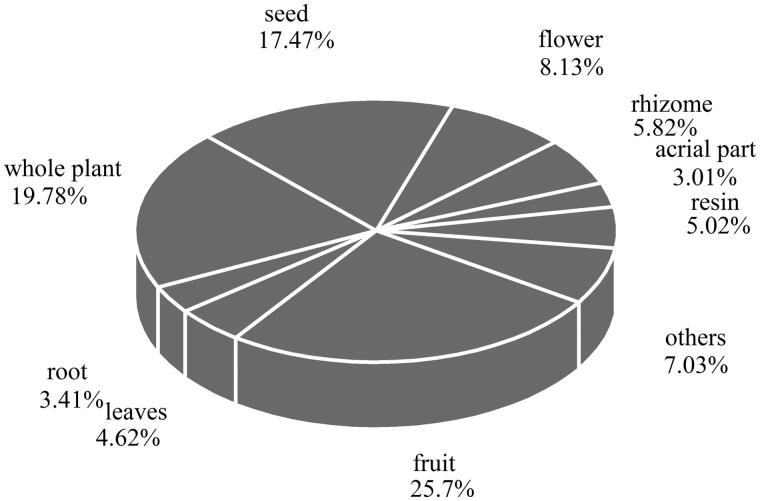
Frequency of aromatic Uygur medicinal plants parts used by the village people of Xinjiang.

Fruits (22 species) were the most commonly used parts of the plants, followed by the whole plant (17 species), seeds (15 species) and flowers (7 species), respectively (Figure 3). Additionally, for 13 species, two or more parts are used in the treatment and curing of diseases, with different parts employed for different effects. For example, the root of *Ephedra* presents a hidroschesis function to treat the night sweats caused by pulmonary tuberculosis and weakness of the body, while the herbaceous stem, which is also used for sweating, is applied to cure colds, coughs, bronchial asthma and malaria. Based on the above findings, we can safely draw the conclusion that different parts of the plants exhibit different functions. We must clarify the function of every part before it can be used to cure diseases (Song et al. 2005).

**Figure 4.**
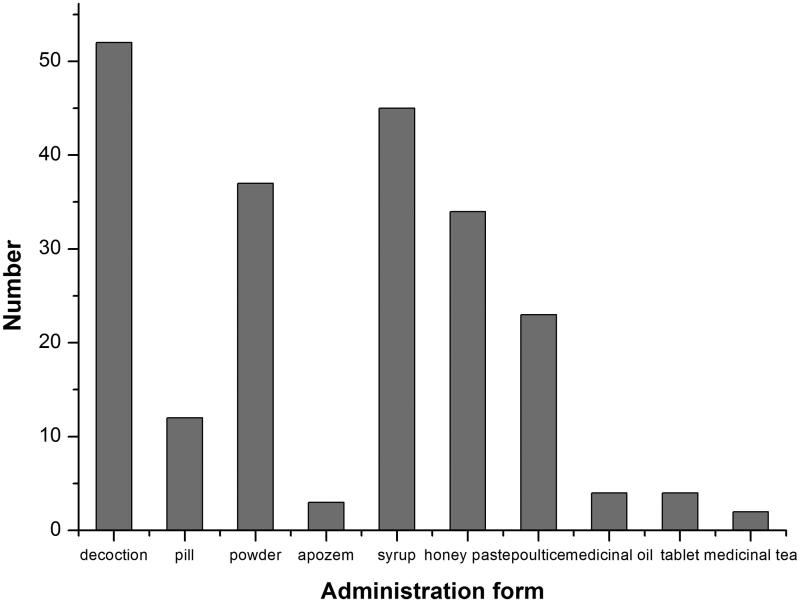
Administration form of aromatic Uygur medicinal plants by village people to treat various ailments.

The results of our survey demonstrated that decoction was the most common mode of preparing aromatic medicinal plants, accounting for 61.72% of the recorded preparations, followed by syrups (47.66%), powders (45.31%), honey pastes (35.16%), poultices (28.13%) and pills (16.41%) (Figure 4). Therefore, there are several methods for the preparation of aromatic medicinal plants (Liu et al. 1993). However, different methods present different efficiencies, and the most appropriate preparation method should be chosen.

### Disorders treated

Based on this survey, the collected aromatic plants are widely used in local traditional Chinese medicine, specifically in Uyghur medicine, to treat gastropathy, liver complaints, parasites and dysentery. Commonly, doctors combine two or more aromatic medicinal plants to treat a particular ailment. In this survey, most of the identified aromatic medicinal plants can be employed as both medicine and food. The local population uses these plants daily to maintain good health in the long-term (Halmurat et al. 2011, King et al. 2015). Some aromatic medicinal plants can be made into healthcare products, such as herbal teas, medicinal liquors and essential oils, which contribute to health in therapies or prevention. In addition, a few of the aromatic plants can be developed into insecticides against parasites. Furthermore, some farmers cultivate aromatic vegetables with certain characteristics that are conducive to supplying the body with necessary nutrients and particular trace elements.

In our survey, plants such as lavender, saffron crocus and mint were found to be commonly used. Lavender essential oil made from lavender plants is good for nervous system disease, paralysis, amnesia, melancholia and arthralgia. Meanwhile it has anti-inflammatory and anti-bacterial functions. The lavender essential oil treatment balanced the inflammatory signaling induced by *S. aureus* by repressing the principal pro-inflammatory cytokines and their receptors and inducing the heme oxygenase-1 gene transcription. The essential oil can stimulate the human innate macrophage response to a bacterium, which is responsible for one of the most important nosocomial infection (Giovannini et al. 2016). Saffron crocus is a kind of common, traditional precious herb among local aromatic medicinal plants. Saffron crocus have anti-oxidant, analgesic, anti-inflammatory, anti-diabetic and several other properties. The kaempferol 3-*O*-rutinoside and kaempferol 3-*O*-glucoside from saffron crocus treatment increased the level of total protein and prevented the carbon tetrachloride-induced increases in serum aspartate aminotransferase, serum alkaline phosphatase and hepatic malondialdehyde levels. And, it has protective effects against acute carbon tetrachloride-induced oxidative liver damage (Wang et al. 2015). Mint presents a wide range of uses; its basic pharmacology involves anti-pyretic and anti-sweating effects. Mint is both a medicinal and culinary herb, employed in mint condiments, spices, teas and so on. There were many aromatic plants identified during this survey that present unique characteristics and play specific roles in the medical community.

### Intake of aromatic medicinal plants

According to the results of our study, the most common methods of application are oral and external, accounting for 72.2% of applications, while 23 of the aromatic plants can be used as oral medicines (26.8%), whereas only one plant, *Nerium indicum* Mill., was reported to be employed only as an externally applied drug (Qian et al. 2005). Under some circumstances, oral and external treatments can better cure disease.

### Additional description of introduced aromatic medicinal materials

#### Families and plant parts

In the present study, some of the medicinal plants we investigated were not native materials. We identified 34 introduced plants, belonging to 24 families, coming from different regions, such as surrounding areas of Europe and the Mediterranean (Souza et al. 2014). Zingiberaceae was the family accounting for the greatest percentage of introduced medicinal materials (25.00%), followed by Rutaceae (20.83%), Lamiaceae (16.67%), Rosaceae (12.50%). Fruits (22.73%) are the most widely used part of the plant, followed by the whole plant (15.91%), roots (13.64%) and seeds (6.82%).

#### Remedy of aromatic plants, administration form and route

Aromatic plants are vital as remedies and in the economic development of Xinjiang. Introduced plants can be used to treat diseases such as colds, gastric diseases and asthma. The most important form of administration of these plants is decoction, similar to findings for native medicinal plants, while the oral administration route is used for every plant. Compared with the native plant species employed in Xinjiang, some introduced plants present specific functions in local use (Di Novella et al. 2013).

## Conclusions

This study first recorded use information on aromatic plants employed in traditional Uyghur medicine in Xinjiang, demonstrating that Xinjiang possesses various raw medicinal herbs. A total of 86 kinds of aromatic plants used by local people belonging to 36 genera were identified, and these plants are still commonly used in daily life. To evaluate the value of the medicinal plants in the target region, the UV was employed in a quantitative analysis. Many plants are used to relieve coughs, eliminate phlegm in treating cardiovascular diseases, colds, haemorrhoids, constipation, stomach diseases, diabetes, urinary diseases, respiratory conditions and throat disease. Therefore, Xinjiang is an area where indigenous medicinal plants present diverse uses, and a sound dimensional medical healthcare treatment system has been developed in this region.

However, some of the traditional Uyghur medicines used in this region still lack physiotherapeutic evidence. Hence, analysis of the chemical constituents and pharmacological activities of certain Uyghur medicines are necessary to explore the potential of Uyghur medicinal plants. This study also provides protection for the local medicinal plant group. Some Uyghur medicinal plants are on the verge of extinction because of frequent natural disasters and the development of urbanization, and the UV of these plants therefore cannot be presented. Thus, the development of further strategies for the conservation of these medicinal plants should be of priority.
